# Transthoracic Echocardiography with Doppler Tissue Imaging predicts weaning failure from mechanical ventilation: evolution of the left ventricle relaxation rate during a spontaneous breathing trial is the key factor in weaning outcome

**DOI:** 10.1186/cc11339

**Published:** 2012-05-14

**Authors:** Sébastien Moschietto, Denis Doyen, Ludovic Grech, Jean Dellamonica, Hervé Hyvernat, Gilles Bernardin

**Affiliations:** 1 Medical Intensive Care Unit, Archet I University Hospital, 151 route Antoine de Ginestière, 06202, Nice, France; 2 Department of Cardiology, Pasteur University Hospital, 30 avenue de la Voie Romaine, 06002, Nice, France

## Abstract

**Introduction:**

There is growing evidence to suggest that transthoracic echocardiography (TTE) should be used to identify the cardiac origin of respiratory weaning failure. The aims of our study were: first, to evaluate the ability of transthoracic echocardiography, with mitral Doppler inflow E velocity to annular tissue Doppler Ea wave velocity (E/Ea) ratio measurement, to predict weaning failure from mechanical ventilation in patients, including those with atrial fibrillation; and second, to determine whether the depressed left ejection fraction and/or diastolic dysfunction participate in weaning outcome.

**Methods:**

The sample included patients on mechanical ventilation for over 48 hours. A complete echocardiography was performed just before the spontaneous breathing trial (SBT) and 10 minutes after starting the SBT. Systolic dysfunction was defined by a left ventricle ejection fraction under 50% and relaxation impairment by a protodiastolic annulus mitral velocity Ea under or equal to 8 cm/second.

**Results:**

A total of 68 patients were included. Twenty failed the weaning process and the other 48 patients succeeded. Before the SBT, the E/Ea ratio was higher in the failed group than in the successful group. The E/Ea measured during the SBT was also higher in the failed group. The cut-off value, obtained from receiver operating characteristics (ROC) curve analysis, to predict weaning failure gave an E/Ea ratio during the SBT of 14.5 with a sensitivity of 75% and a specificity of 95.8%. The left ventricular ejection fraction did not differ between the two groups whereas Ea was lower in the failed group. Ea increased during SBT in the successful group while no change occurred in the failed group.

**Conclusions:**

Measurement of the E/Ea ratio with TTE could predict weaning failure. Diastolic dysfunction with relaxation impairment is strongly associated with weaning failure. Moreover, the impossibility of enhancing the left ventricle relaxation rate during the SBT seems to be the key factor of weaning failure. In contrast, the systolic dysfunction was not associated with weaning outcome.

## Introduction

Cardiogenic pulmonary edema has been recognized as a frequent cause of weaning failure [1-3]. The definitive diagnosis remains difficult; in this situation right heart catheterization was proposed as the reference tool [3,4]. Lemaire *et al*. demonstrated that a spontaneous breathing trial (SBT) induced an elevation in the left ventricular filling pressure (LVFP), which plays a key role in weaning failure [3]. Since the introduction of a pulmonary artery catheter remains an invasive and potentially harmful procedure, there is growing evidence to suggest that transthoracic echocardiography (TTE) should be used to identify the cardiac origin of respiratory weaning failure [4]. Tissue Doppler is a novel technique that directly measures myocardial velocities [5,6]. The early diastolic mitral annulus velocity (Ea) has been shown to be a relatively load independent measure of myocardial relaxation [6-8]. When Ea is combined with pulsed Doppler mitral flow in early diastole E, the resulting E/Ea ratio closely correlated with the measured invasive LVFP [6,9]. Lamia *et al*. showed that the combination of E/A mitral inflow and E/Ea at the end of a SBT predicted an increase in the LVFP, with a good sensitivity and specificity, in a selected population of patients who were difficult to wean [4]. More recently, Caille *et al*. found a significantly higher E/Ea ratio at baseline in failed patients [10]. These studies excluded patients with atrial fibrillation, which represent 20 to 30% of patients hospitalized in intensive care units [11]. Nevertheless, despite this arrhythmia, the LVFP could be assessed accurately with measurement of the E/Ea ratio [12].

The primary objective of our study was to test the ability of the mitral E/Ea ratio, measured at baseline and shortly after initiation of SBT, to determine the risk of weaning failure in a non-selected population, including patients with atrial fibrillation. The second aim was to determine whether the depressed left ejection fraction and/or diastolic dysfunction participated in weaning outcome.

## Materials and methods

This observational prospective study was conducted from November 2009 to April 2011 in a 10-bed medical intensive care unit. No change of standard care was introduced for the needs of this study, which was, therefore, accepted as a descriptive study by the clinical research committee of the university hospital of Nice, in accordance with the ethical standards laid down in the 1964 Declaration of Helsinki. Because it is a descriptive study, written informed consent was waived but all patients involved in our study or their next of kin were informed and accepted to participate.

We screened patients under mechanical ventilation for more than 48 hours before they were considered ready to undergo an initial SBT. Patients were included if they met the following criteria: improvement of the underlying cause of acute respiratory failure, body temperature < 39°C, hemoglobin level > 7G/dl, PaO2 > 60 mmHg, FIO2 < 40%, positive end expiratory pressure (PEEP) under or equal to 8 cm H2O, respiratory rate less than 35 breathes/minute, systolic arterial pressure > 90 mmHg and < 160 mmHg without need for vasoactive drugs, no sedation and a stable neurological status [13].

Non-inclusion criteria included tracheostomy, active neuromuscular disease, pregnant women, a poor echocardiography window, heart rate faster than 140 beats per minute at baseline, and patients with atrioventricular conduction abnormalities. Patients with severe mitral regurgitation, mitral stenosis or a prosthetic mitral valve were also excluded because E/Ea had not been validated in such patients for evaluation of LVFP.

### Spontaneous breathing trial

The SBT lasted one hour and consisted of a low pressure support trial at 7 cm H2O without PEEP [14]. Patients were considered to have failed the SBT if they developed any of the following signs during the one-hour SBT [15]: respiratory frequency > 35 breaths per minute, arterial oxygen saturation < 90%, heart rate > 140 beats per minute or sustained increase or decrease in blood pressure with systolic arterial pressure > 200 or < 80 mmHg, diaphoresis and signs of distress. The time of occurrence of these signs after starting the SBT was noted. Patients who succeeded the SBT were extubated. The attending physician in charge of the patient had no access to the TTE findings. Failure of the weaning process was defined as a failed SBT or the need for reintubation within 48 hours following extubation. Diagnosis of weaning failure was carried out by two intensivists blinded to the TTE report. Based on clinical and radiological findings they confirmed or excluded the diagnosis of cardiogenic pulmonary edema. The following signs or information were used and were assumed to be suggestive of weaning induced pulmonary edema: previous history of heart disease, exclusion of other causes of respiratory failure, early onset of respiratory distress after suppression of PEP, the presence of frothy secretions and bilateral crackles and onset of newer bilateral infiltrates on chest x-ray.

### Bedside monitoring

During assisted mechanical ventilation, the level of pressure support (AI), PEEP, the patient's respiratory rate (RR), tidal volume (VT) and the RR/VT ratio [16] were recorded just before initiation of the SBT. Arterial blood gas analysis was performed just before and at the end of the SBT. The heart rate, respiratory rate, blood pressure, pulsed oximetry and consciousness were monitored throughout the SBT.

### Bedside echocardiography

TTE was performed using a Vivid 7 General Electric^® ^healthcare Amersham United Kingdom just before the SBT, and 10 minutes after starting the SBT. The left ventricular ejection fraction (LVEF) was assessed using Simpson's method. Pulsed-wave Doppler analysis of mitral inflow allowed measurement of the E wave velocity and the E wave deceleration time (DTE). Pulsed wave Doppler tissue imaging (DTI) was performed at transducer frequencies of 3.5 MHz with an as possible optimal gain, to obtain the best signal to noise ratio. The myocardial velocity was recorded using the DTI technique. The sample volume was placed at the junction of the left ventricular wall with the mitral annulus of the lateral myocardial segments from apical four-chamber views. Ea was measured and E/Ea was then calculated. The mean of three measurements was used for calculation. All the Doppler flow measurements were performed at the end of the expiratory period. In the case of atrial fibrillation, E/Ea was averaged over 10 cardiac cycles [12].

Systolic dysfunction was defined by a LVEF below 50%. Relaxation impairment was confirmed by a velocity Ea below or equal to 8 cm/sec [17]. Cardiac ischemia during SBT was identified by new left ventricular (LV) segmental wall motion abnormalities. All the echocardiographic examinations were performed by a cardiologist blinded to the clinical outcome.

### Statistical analysis

Statistical analysis was performed using IBM SPSS^® ^Software 19.0.0 Chicago, United states of America. Continuous variables were expressed as medians and interquartiles (25^th ^to 75^th ^percentile), and categorical variables as percentages. Categorical variables were analyzed with a Chi-square test or the Fisher's exact test. Continuous variables were compared using the U Man and Whitney or the Wilcoxon test as appropriate. Two-tailed *P-*values less than 0.05 were considered statistically significant. Receiver operating characteristics (ROC) curve analysis was performed to assess the ability of E/Ea to predict weaning failure. To examine inter-observer variability, a co-investigator, blinded to the clinical information and to the results of the first investigator, examined 10 randomly selected Doppler mitral inflow and Doppler tissue images to determine E/Ea. Inter-observer variability was calculated as the difference between the values obtained by the two observers divided by the mean.

## Results

From November 2009 to April 2011, 82 patients were screened, but 14 were excluded for the following reasons: suboptimal echocardiography images (*n *= 7), neuromuscular disease (*n *= 3), and tracheostomy (*n *= 4).

Sixty-eight patients were included. The reasons for intubation were septic shock (*n *= 20), cardiac arrest (*n *= 5), cardiogenic pulmonary edema (*n *= 6), acute respiratory distress syndrome (*n *= 10), post-operative period (*n *= 7) pneumonia (*n *= 12) and coma (*n *= 8).

Twenty patients failed to wean. Sixteen patients failed the SBT, 14 patients with cardiogenic pulmonary edema and 2 with respiratory acidosis without hypoxemia. The cause of these two respiratory failures was under diagnosed ICU acquired weakness. These two patients underwent tracheotomy, one died in intensive care while the other one was delivered from mechanical ventilation four weeks later.

Four patients failed in the post extubation period. The time of occurrence of SBT failure was 24 minutes [17-33]. The reasons for extubation failure were septic shock (*n *= 2) and excessive bronchorrhea (*n *= 2).

Fifteen patients presented with atrial fibrillation (22.1%) at the time of inclusion, among these 15 patients 9 (60%) failed the SBT and all presented with weaning induced pulmonary edema. Six others patients succeeded the SBT and none needed re-intubation.

### Clinical characteristics

Compared to the successful group, there was significant greater intensive care unit mortality in patients who failed the weaning process (35% versus 8.3%, *P *= 0.05; Table 1). Diabetes mellitus was more frequent in the failed group (30% versus 8.3%, *P *= 0.05; Table 1).

**Table 1:** Clinical characteristics of patients

	Weaning success *n *= 48	Weaning failure *n *= 20	*P*
Age (years)	66 (59 to 73)	68 (62 to 72)	0.4
Gender (% male)	30 (62.5)	9 (45)	0.28
SAPS II score	54 (48 to 62)	51 (45 to 55)	0.23
Duration of mechanical ventilation (days)	5.0 (3.0 to 8.5)	9.0 (4.0 to 12.0)	0.06
ICU mortality (%)	4 (8.3)	7 (35.0)	0.05
Reasons for intubation			
Cardiac arrest	4 (8.3)	1 (5.0)	
ARDS (%)	7 (14.5)	3 (15.0)	
Septic shock (%)	14 (29.1)	6 (30.0)	
Pneumonia (%)	10 (20.8)	2 (10.0)	
Cardiogenic pulmonary edema (%)	4 (8.3)	2 (10.0)	
Coma	6 (12.5)	2 (10.0)	
Surgery	3 (6.2)	4 (20.0)	
Patients in atrial fibrillation (%)	9 (18.7)	6 (30.0)	0.48
COPD history (%)	9 (18.7)	6 (30.0)	0.2
Diabete mellitus	4 (8.3)	6 (30.0)	0.05
Arterial hypertension	19 (39.6)	14 (70.0)	0.04
Plasma creatinine (micromol/l)	87 (57 to 203)	102 (53 to 157)	0.56
Hemoglobin (g/dl)	10.9 (8.7 to 11.4)	9.8 (9.5 to 10.5)	0.65
Relaxation impairment, n (%)	17 (35.4)	16 (80)	0.0004
Systolic dysfunction, n (%)	9 (18.7)	4 (20.0)	> 0.99
LVEF (%)	55 (50 to 60)	60 (50 to 65)	0.2

Data are medians (25^th ^to 75^th ^percentile) or numbers (% of patients). ARDS, Acute Respiratory Distress Syndrome; COPD, Chronic Obstructive Pulmonary Disease; LVEF, Left Ventricle Ejection Fraction; SAPS II, Simplified Acute Physiologic Score

The LVEF did not differ significantly between groups, whereas the prevalence of left ventricular relaxation impairment was higher in the failed group (Table 1).

### Baseline characteristics

Patients in the failed group had a higher respiratory rate, a lower VT and a higher RR/VT ratio (Table 2). Before SBT, Ea was lower and E/Ea was higher in the failed group.

**Table 2:** Clinical, biological and echocardiographic parameters measured before and during spontaneous breathing trial

	Baseline			Spontaneous breathing trial		
	Success of weaning *n *= 48	Weaning failure *n *= 20	*P*	Success of weaning *n *= 48	Weaning failure *n *= 20	*P*
Mean blood pressure (mmHg)	132 (122 to 144)	135 (125 to 135)	0.91	145 (131 to 155)	175 (149 to 180)	0.0007
Heart rate (beats/min)	93 (79 to 106)	98 (82 to 105)	0.75	102 (87 to 110)	120 (106 to 122)	0.002
Inspiratory pressure support (cm H_2_O)	11 (10 to 12)	12 (10 to 13)	0.17	7 (7 to 7)	7 (7 to 7)	> 0.99
PEP (cm H_2_O)	5 (5 to 5)	5 (5 to 5)	0.45	0 (0 to 0)	0 (0 to 0)	> 0.99
Respiratory rate (breaths/min)	20 (16 to 25)	28 (24 to 30)	0.0008	23 (19 to 28)	45 (39 to 49)	< 0.0001
RR/VT	46 (26 to 58)	66 (55 to 78)	0.0005	50 (30 to 66)	136 (130 to 156)	< 0.0001
Arterial pH	7.45 (7.41 to 7.48)	7.45 (7.42 to 7.5)	0.46	7.45 (7.41 to 7.48)	7.43 (7.38 to 7.49)	0.27
PAO_2_/FIO_2_	260 (235 to 303)	214 (206 to 253)	0.02	227 (211 to 254)	167 (149 to 200)	0.0002
PCO_2 _(mmHg)	37 (33 to 42)	38 (36 to 43)	0.29	37 (34 to 41)	43 (37 to 50)	0.005
E wave (cm/sec)	72 (62 to 91)	80 (67 to 108)	0.14	80 (69 to 107)	105 (91 to 125)	0.007
DTE (msec)	215 (179 to 266)	170 (160 to 223)	0.03	181 (150 to 205)	155 (128 to 180)	0.04
Ea (cm/sec)	8 (7 to 10)	7 (6 to 8)	0.01	10 (8 to 12)	7 (6 to 8)	0.0003
E/Ea	8.9 (7.2 to 11.3)	13.4 (8.5 to 16.4)	0.001	8.4 (7.1 to 11.6)	15.7 (13.4 to 21.1)	< 0.0001

Data are medians (25^th ^to 75^th ^percentile) or numbers (% of patients). DTE, deceleration time of E wave; Ea, protodiastolic annulus mitral velocity; E wave, protodiastolic mitral velocity; PAO2/FIO2, Pressure Arterial Oxygen/Fraction Inspired Oxygen; PEP, Positive Expiratory Pressure; RR/VT, Respiratory rate/Tidal Volume

### Parameters measured during the SBT

Ea was still lower and E/Ea higher in the failed group (Table 2). One patient in the failed group presented with myocardial ischemia during SBT, as attested by renewed septal dyskinesia

### Parameter changes during the SBT

**Figure 1:**
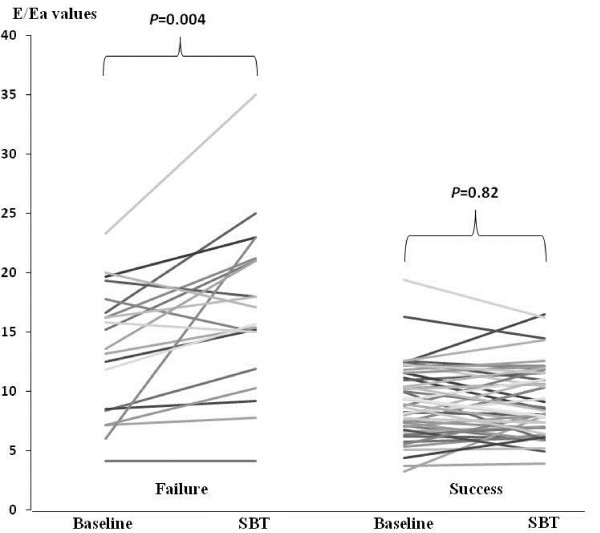
**Individuals values of E/Ea mitral ratio in the failure and the success group**. This figure represents individual values of E/Ea measured at baseline and during SBT for patients in failure group **(A) **and for patients in the success group **(B)**. Significant increase in E/Ea occurs during SBT in the failure group while no variation in E/Ea is observed in the success group.

**Figure 2:**
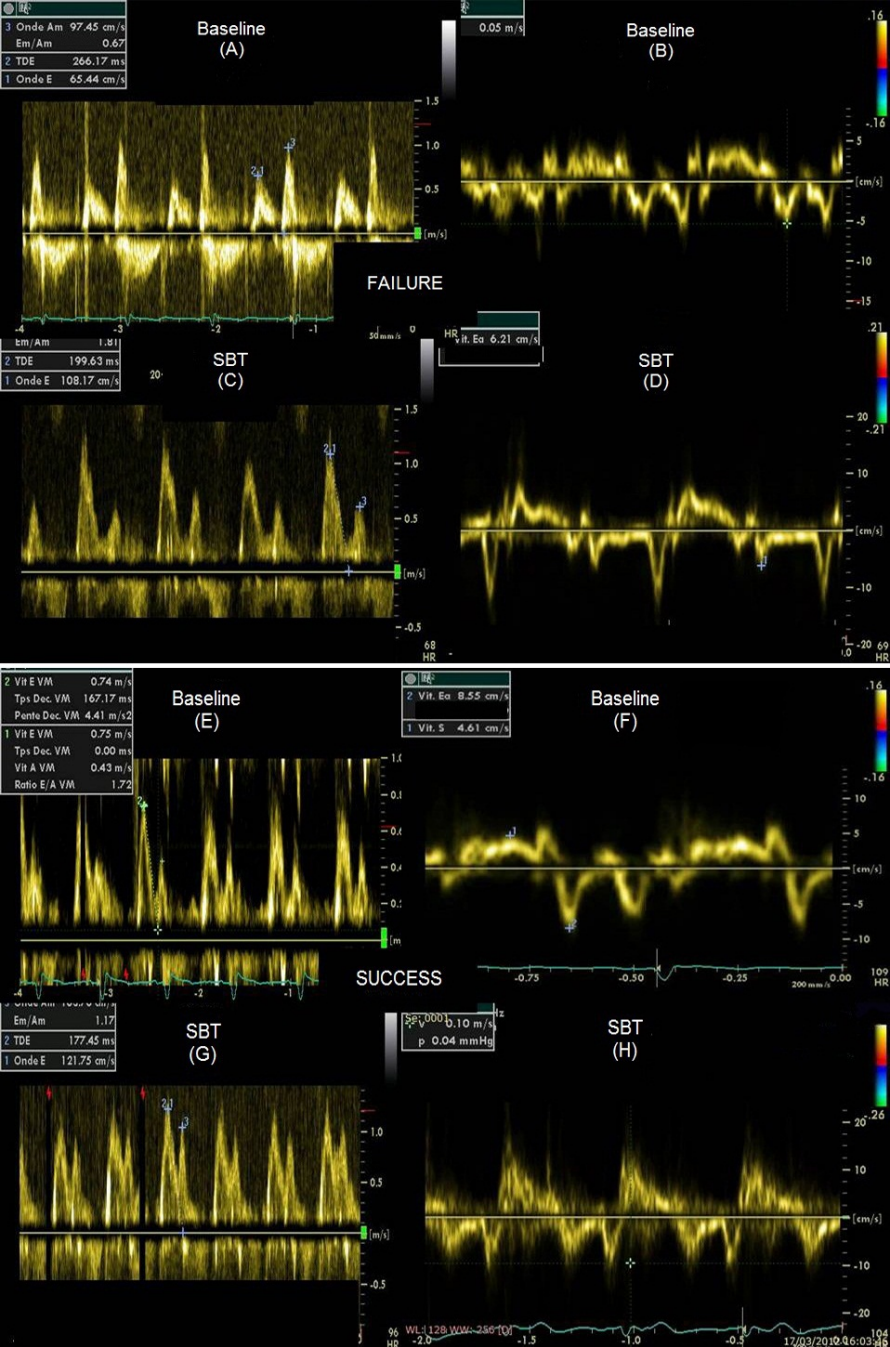
**Doppler figure with E/Ea measurement at baseline and during SBT in success and failure**. **(A) **Represents tissue Doppler of mitral annulus at baseline, amplitude of early diastolic wave Ea is 5 cm/sec, the resultant E/Ea ratio is 13. **(B) **E increases during SBT to 108 cm/sec. **(C) **Ea is 6.2 cm/sec, the resultant E/Ea increases during SBT to 17.4. **(D) **Represents pulsed Doppler of mitral flow at baseline in a patient who succeeded, E is 74 cm/sec. **(E) **Ea is 8.5 cm/sec, the resultant E/Ea is 8.7. **(F) **E increases during SBT to 121 cm/sec. **(G) **In the same way Ea increases to 10 cm/sec, then E/Ea ratio remains low during SBT at 12.1.

The SBT was accompanied by changes in the echocardiographic index (Figures 1 and 2). A significant increase in the E/Ea ratio was observed in the failed group (13.4 (8.4 to 16.3) versus 15.7 (13.4 to 21.1), *P *= 0.004), while no variation was observed in the successful group (8.9 (7.2 to 11.3) versus 8.4 (7.1 to 11.6), *P *= 0.82). No significant variation of Ea was observed in the failed group (7 (6 to 8) versus 7 (6 to 8), *P *= 0.9), whereas LV relaxation was enhanced in the successful group with an increase in Ea velocity (8 (7 to 10) versus 10 (8 to 12), *P *< 0.001).

### Predictive value of the E/Ea ratio

**Figure 3:**
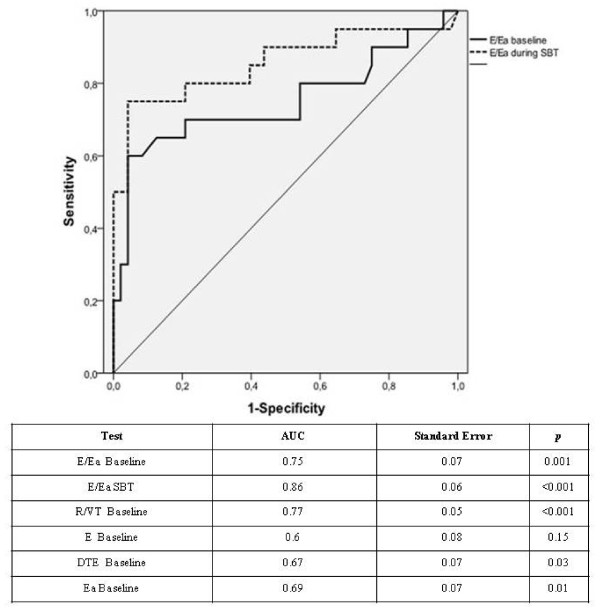
**ROC curves for E/Ea measured at baseline and during the SBT to predict weaning failure**. This figure shows ROC curves for E/Ea measured before SBT in solid line and E/Ea measured 10 minutes after starting the SBT in dashed line to predict weaning failure. AUC for protodiastolic annulus mitral velocity (Ea), Protodiastolic mitral velocity (E) wave its deceleration time (DTE) and ratio of respiratory rate/Tidal volume (RR/VT) are summarized in the table under ROC curves. AUC, Area under the ROC curve.

The area under the ROC curve for the E/Ea ratio measured at baseline in predicting weaning failure was 0.75 (± 0.07, *P *= 0.009). A cut-off value more or equal to 12.6 was associated with the highest diagnostic accuracy and predicted weaning failure with a sensitivity of 60% and a specificity of 95.8%; it provided a positive likelihood ratio value of 14.4 and a negative likelihood ratio of 0.42. E/Ea measured 10 minutes after starting the SBT had a greater predictive value, with an area under the ROC curve of 0.86 (± 0.06, *P *< 0.001). The cut-off value of 14.5 was associated with the highest diagnostic accuracy. This ratio predicts weaning failure with a sensitivity of 75% and a specificity of 95.8%. It provided a positive likelihood ratio value of 18.0 and a negative likelihood ratio of 0.26 (Figure 3).

Reproducibility of E/Ea measurement was satisfactory with an inter-observer variability of 3.4% (1.7 to 6.5%).

### Predictive value of the E/Ea ratio in patients with atrial fibrillation

We also analyzed the diagnostic accuracy of E/Ea in predicting weaning failure in the subgroup of patients with atrial fibrillation. The area under the ROC curve for E/Ea at baseline in predicting weaning failure was (0.87 +/- 0.12, *P *= 0.02) and the area under the ROC curve for E/Ea during SBT in predicting weaning failure was (0.92 +/-0.07, *P *= 0.007).

### Changes in the E/Ea ratio between consecutive SBT

In 12 patients who had failed the first SBT, E/Ea was measured again during a second SBT. Between the two trials, 10 patients received diuretics and 2 received the angiotensin-converting enzyme inhibitor. Nine patients succeeded this second weaning trial and three failed. E/Ea decreased in the late successful group (15.4 (11.9 to 21) versus 11 (8.9 to 13.5), *P *= 0.01) but not in the late failed group (23 (22.1 to 24.0) versus 23 (21.9 to 23.9), *P *= 0.18).

## Discussion

The major finding of this study shows that serial measurement of E/Ea accurately predicts weaning failure. E/Ea prior to SBT was significantly higher in the failed group; however, diagnosis in predicting weaning failure, as estimated from the ROC curve was poor. Interestingly, E/Ea measured 10 minutes after starting the SBT showed better diagnostic performance; the cut-off value of 14.5 predicted weaning failure with a sensitivity of 75% and a specificity of 95.8%.

Moreover, the TTE detected changes in the central hemodynamics induced by the SBT. The failed group showed a marked increase in E/Ea, whereas no significant change was observed in the successful group. These results are suggestive of an increase in LVFP during the SBT in the sole failed group. These findings are in line with previous studies; pulmonary artery catheterization showed an increase in the pulmonary artery occlusion pressure (PAOP) during the SBT, only in patients who failed the weaning process [18].

In our study, no significant difference in the E mitral was observed at baseline between the two groups. This is explained by the fact that the E velocity or even E/A is not only determined by LVFP but also depends on ventricular factors, such as the relaxation rate, age of patients, compliance and systolic function [19]. Correlation between E/A mitral and LVFP were too weak in giving an accurate estimation [5]. The E/A mitral should not be used alone and must be considered in the context of the clinical picture, especially with respect to age and systolic function. These difficulties in interpreting the E/A ratio limit its use in clinical practice. In contrast, E/Ea closely correlated with the measured invasive LVFP, even in patients under mechanical ventilation [9].

As in our study, Caille *et al*. found a significantly higher E/Ea prior to SBT in patients who fail, but their cut-off values for E/Ea were lower than in the present study [10]. This discrepancy is explained by a gray zone when using E/Ea alone for the estimation of the LVFP. Ommen *et al*. showed that an E/Ea < 8 accurately identified patients with a normal LVFP, and E/Ea > 15 for those with an elevated LVFP [20]. For patients with intermediate values of E/Ea, between 8 and 15, it can be difficult to accurately estimate the LVFP using only E/Ea [20]. Lamia *et al*. demonstrated that the combination of E/A > 0.95 mitral and E/Ea > 8.5 predicted an elevation in PAOP at the end of the SBT and could then identify patients at risk of weaning failure [4]. Nevertheless, patients with atrial fibrillation exhibited a single mitral flow. Some patients present with partial or complete merging of the E and A wave due to tachycardia, despite a normal sinus rhythm. E/A could not be used in these cases and this limits its utilization for clinical practice at the bedside. Nagueh *et al*. demonstrated that E/Ea could be used to assess the LVFP with good accuracy in sinus tachycardia [21], even with complete merging of the E and A waves, and in the same way in patients with atrial fibrillation [12]. Patients with atrial fibrillation cannot be excluded from this study because they represent 20 to 30% of patients hospitalized in intensive care unit. Our study is the first to include such patients. For all these reasons, E/Ea seems to be the measure of choice to predict weaning failure at the bedside, especially in those with a preserved or slightly reduced LVEF, atrial fibrillation or in those with single mitral flow due to sinus tachycardia.

The second major finding of this study shows that the systolic function assessed by LVEF was not associated with weaning outcome. This is in accordance with previous studies. Mekontso *et al*. [22] showed, in a subgroup of patients who underwent TTE, that the LVEF did not differ between patients in the failed or in the successful group. Similar data were obtained by Lamia *et al*. [4] and more recently by Zapata *et al*. [23]. This apparent paradox is explained by the fact that in studies into cardiology, 40 to 50% of patients with typical signs of heart failure have a normal or slightly reduced LVEF [24-27]. So the LVEF is not a good predictor of clinical disability and suggests that congestive symptoms are more closely related to the diastolic properties of the ventricle than to the systolic properties [24,25]. The diagnosis of diastolic abnormalities can be confirmed when Doppler with tissue imaging provides evidence of impaired myocardial relaxation [7,28,29]. The Ea velocity constitutes a non-invasive and relatively load independent index of LV relaxation and diastolic function. Relaxation impairment is defined by an Ea velocity below or equal to 8 cm/sec [7]. In our study, in comparison to the successful group, patients in the failed group had a lower Ea velocity and exhibited more frequent impairment in relaxation. Abnormal relaxation resulted in a prolongation of the isovolumic relaxation time with an upward shift and to the left of the diastolic pressure/volume curve (Figure 2) [24]. In such patients, a relatively small increase in the end diastolic volume may lead to an exaggerated increase in LVFP despite a normal end diastolic volume [24]. This increased sensitivity to volume changes could explain the occurrence of pulmonary edema during the SBT because an inspiratory drop in the intra-thoracic pressure tends to increase the systemic venous return and LV preload.

In our study, the evolution of the relaxation rate was also important. The failed group showed a reduced Ea before the SBT, and the Ea did not vary during the SBT, whereas in the successful group the Ea increased during the SBT. This finding explains why E wave increased during SBT in both groups. This change in mitral flow reflects an enhancement in the LV relaxation rate for the successful group while it reflects a major increase in LVFP for the failed group [30]. This is in agreement with previous studies that demonstrated that in healthy individuals the Ea velocity increased with exercise; however, in patients with abnormal relaxation and a reduced Ea velocity, the Ea did not vary with exercise or with loading conditions [31-33]. During exercise, like during the SBT, the LV filling rate could increase dramatically [31]. Normally, this increased filling occurred because the left ventricle early diastolic pressure decreased with enhanced myocardial relaxation. Thus a substantial increase in filling could occur without an increase in LVFP [32]. This normal response to exercise was lost in heart failure in patients with diastolic dysfunction who were not able to enhance relaxation with exercise [32]. In patients with impairment in relaxation, increases in LV filling in response to exercise were dependent on increases in LVFP [32]. Because the cardiovascular response to weaning from mechanical ventilation is similar during exercise [34], these considerations could explain the marked increase in LVFP in patients with diastolic dysfunction during the SBT. To our knowledge, this is the first study demonstrating that relaxation impairment and the inability to enhance LV relaxation during the SBT were closely associated with the risk of weaning failure.

Our study holds several limitations. In our study, diastolic dysfunction was associated with weaning failure; however, we included a large proportion of patients with chronic obstructive pulmonary disease [35], diabetes [36], hypertensive history, and atrial fibrillation [37]; all these clinical disorders are known to be associated with diastolic dysfunction. At present, this particular population precludes generalization of our findings to all populations of intensive care units. Another limitation is the poor echocardiographic windows in some patients under mechanical ventilation; seven patients were excluded because of suboptimal images.

The diagnosis of weaning-induced pulmonary edema was made using a clinical approach; we did not assess invasive PAOP. However, the diagnosis of cardiac dysfunction during weaning is still difficult and the clinical presentation is often non-specific. In contrast, the diagnosis of weaning failure is clearly defined; this is why we chose to evaluate the utility of serial measurement of E/Ea to predict weaning failure and not the risk of cardiac dysfunction.

## Conclusions

Transthoracic echocardiography with measurement of the E/Ea ratio can identify patients at risk of weaning failure at the bedside. A reduced LVEF did not seem to influence the weaning outcome, whereas diastolic abnormalities with impaired relaxation and no ability to enhance relaxation during the SBT are closely associated with weaning failure.

## Key messages

• Transthoracic echocardiography with E/Ea mitral ratio predicts weaning failure at bedside even in patients with atrial fibrillation

• Left ventricle ejection fraction is not associated with weaning failure

• Diastolic properties, especially left ventricle relaxation impairment, is closely associated with weaning outcome

• The impossibility of enhancing the left ventricle relaxation rate during spontaneous breathing trial seems to be the key factor of weaning failure.

## Abbreviations

DTE: E wave deceleration time; DTI: Doppler tissue imaging; E/Ea: mitral Doppler inflow E velocity to annular tissue Doppler Ea wave velocity; Ea: early diastolic mitral annulus velocity; LV: left ventricular; LVEF: left ventricular ejection fraction; LVFP: left ventricular filling pressure; PAOP: pulmonary artery occlusion pressure; PEEP: positive end expiratory pressure; ROC: receiver operating characteristics; RR: respiratory rate; SBT: spontaneous breathing trial; TTE: transthoracic echocardiography; VT: tidal volume

## Competing interests

The authors declare that they have no competing interests.

## Authors' contributions

SM and DD conceived the study protocol. SM, DD, LG, JD, HH and GB participated in the design and coordination of the study. SM and DD drafted the present manuscript. All authors read and approved the final manuscript.
